# Role of catalytic nitrile decomposition in tricopper complex mediated direct partial oxidation of methane to methanol

**DOI:** 10.1038/s41598-021-98721-2

**Published:** 2021-09-28

**Authors:** Ehsan Moharreri, Tahereh Jafari, Dinithi Rathnayake, Harshul Khanna, Chung-Hao Kuo, Steven L. Suib, Partha Nandi

**Affiliations:** 1 Department of Chemistry and Institute of Materials Science, University of Connecticut, Storrs, CT 06269 USA; 2 Corporate Strategic Research, ExxonMobil Research and Engineering, 1545 US 22 East, Annandale, NJ 08801 USA

**Keywords:** Catalysis, Inorganic chemistry, Organic chemistry

## Abstract

Synthetic homogeneous system known to date performing methane to methanol conversion using O_2_ as terminal oxidant is unique and based on copper complex with piperazine-based ligand (Cu_3_L in Fig. 1) in a medium of acetonitrile. Prior work have shown that in order to achieve catalytic turnover, hydrogen peroxide is needed to regenerate the active site. We show in this paper that reaction solvent based on organic nitrile decompose concurrently with methane activation and that in the absence of either acetonitrile, Cu complex or hydrogen peroxide, the catalytic turnover does not happen. We show in this manuscript that the direct methane oxidation to methanol might have been mediated by catalytic Radziszewski oxidation between acetonitrile and H_2_O_2_. Additionally we have discovered that in the absence of methane, peroxide mediated acetonitrile decomposition also makes methanol via a background reaction which was hitherto unknown.

## Introduction

Methane monooxygenase serves as a model to develop biomimetic complexes to turnover methane to methanol under mild conditions. In a remarkable study, Chan et al. introduced the Cu_3_LO_2_ complex made after pMMO active site^1–3^. Mechanistic differences in the Cu_3_LO_2_ complex^3^ and Cu-oxo clusters in zeolites are still unresolved^4–6^. Chan et al., showed that in the absence of methane, the use of O_2_ and H_2_O_2_ as oxidant and reductant could lead to an abortive cycle^7^. The supported version of this catalyst exhibited 171 turnover number when using nearly 200 equivalents of H_2_O_2_ in the presence of acetonitrile^8^. Although the supported hybrid organic–inorganic Cu complex showed a high degree of selectivity, the system did not exhibit catalytic turnover using O_2_ as terminal oxidant. We had tried H_2_ + O_2_, Au–Pd/TiO_2_ system^9^ for generating hydrogen peroxide in situ along with Cu_3_L complex where we saw almost no methanol formation with water as the solvent. We had also looked at tert-Butyl hydroperoxide (TBHP) which did not succeed as a reducing agent for Cu_3_L regeneration. Given that the Cu_3_L complex does neither turnover with O_2_, nor in aqueous solution we sought to further probe the role of acetonitrile in this catalytic mechanism^3^. The Radziszewski oxidation^10,11^ proceeds via nucleophilic addition of hydroperoxide anion to an organic nitrile carbon to afford peroxyimidate which spontaneously undergoes a rearrangement reaction to release singlet oxygen and amide^12^. The evolution of reactive oxygen species from this nitrile decomposition is used for affording selective partial oxidation of a number of functional groups such as sulfoxidation^13,14^, epoxidation^15^, and Bayer–Villiger oxidation^16^. Peroxide and acetonitrile combination in the presence of metals catalyze the partial oxidation of alkanes^17–20^. Complexes of Cu(II) along with H_2_O_2_ result in producing O_2_ and OH^**.**^ radicals via a Cu(II)/Cu(I) redox cycle^21^. Copper catalyzed decomposition of H_2_O_2_ is well studied through Fenton like autocatalysis^22^, alkaline solutions^23^, and Cu(I)/Cu(II) and Cu(II)/Cu(III) redox pairs^24^. We probed the rate of Cu(I) incorporation to form the Cu_3_L framework. Ligand structure controls the copper oxygen bonding type^25^ and substrate angle by steric effects^3,26^. UV–Vis data indicates counter anions^27^ can alter the redox properties as indicated by a shift in the charge transfer bands for bidentate ligands^28^ with fewer coordinating anions leading to bis(µ-oxo)dicopper(III) and more strongly coordinating anions leading to peroxodicopper(II). Spectroscopic methods are capable of determining the nature of copper oxygen bonding species^29^. ESI–MS analysis is used to show qualitative purity of Cu_3_L complex species in a solution^30^. There are instances of acetonitrile and hydrogen peroxide environments for C–H bond activation where solvent decomposition and generation of reactive oxygen species through acetonitrile oxidation has not been investigated^31–38^. Palomas et al., used multicopper complexes for methane oxidation which was rather selective towards CO_2_^39^. In all instances of such complexes showing activity, there was a medium of peroxide and nitriles present^1,3,7,40–42^. Previously the role of acetonitrile as a mediator for oxygen transfer was not known. Here we further elucidate mechanism by independently assessing various organic nitrile decomposition with peroxide.

## Results

**Figure 1:**
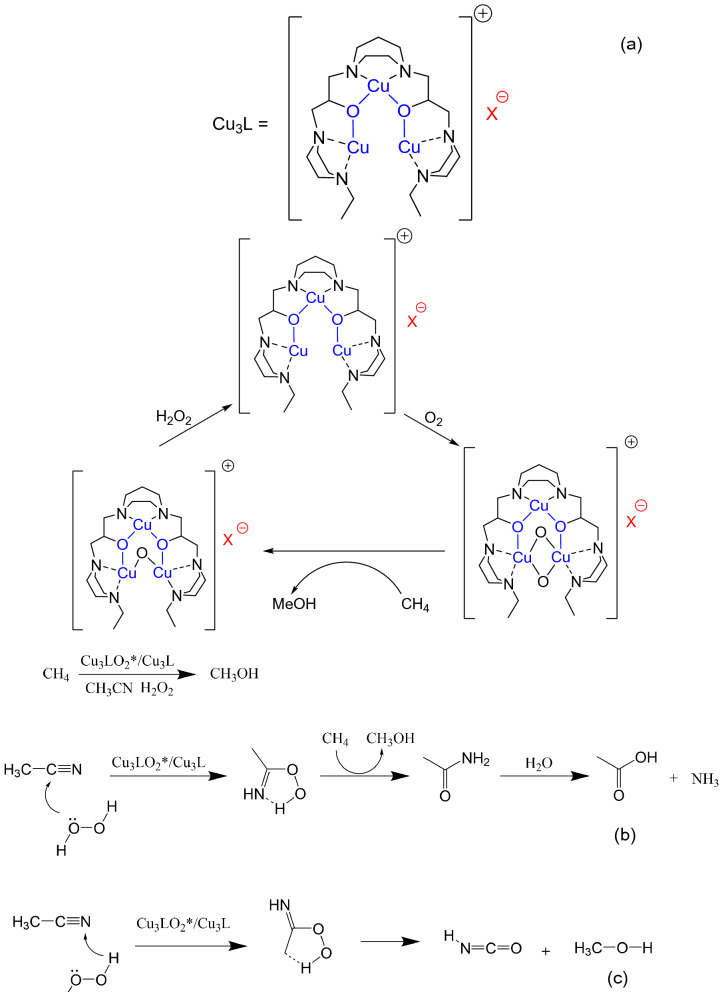
Proposed mechanism of tricopper complex mediated acetonitrile oxidation. (**a**) Shows prior work while (**b**) is this work’s findings on acetonitrile decomposition and (**c**) methanol and formamide (via isocyanurate hydration) production from acetonitrile.

Ligand 3,3′-(1,4-diazepane-1,4-diyl)bis[1-(4-ethylpiperazine-1-yl)propan-2-ol] (7-N-Etppz) and copper incorporation into this complex is controlled by time and ligand ratio as indicated by the ESI–MS stacked plot (Supplementary Fig. 2–3). Acetonitrile attaches to the complex in nearly all of the tricopper complex species Supplementary Fig. 2–4. More tricopper species are formed with acetate and perchlorate than with tetrafluoroborate, exemplified by Supplementary Table 1. The ligand species is indicated by 441 *m/z*, and 503 *m/z* is the monocopper incorporated species. When the ratio of copper to ligand is around 3, the ligand fully incorporates into the complex (Supplementary Fig. 3). The spectroscopic characteristics are summarized in Supplementary Table 2. Characterization spectroscopically helps determine copper species as summarized in EPR Table Supplementary Table 3. Addition of hydrogen peroxide led to hyperfine coupling features in EPR hence less interactive Cu^II^ core, while the oxidized Cu_3_LO_2_ complex has broad features indicative of closely interacting Cu^II^ core. The complex with BF_4_ precursor does not show signal prior to oxidation since all is in Cu^I^ form. UV–Vis characterization of oxidized (Supplementary Fig. 5) and H_2_O_2_ treated (Supplementary Fig. 6) indicates closed complex and integrated clusters with Acetate and ClO_4_ counter anions. H_2_O_2_ addition reduces the oxide-to-Cu^II^ LMCT. Since nitrile coordinates towards copper site in the complex, it becomes susceptible to nucleophilic attack. Figure 1 and Supplementary Table 4 summarize the Radziszewski acetonitrile partial oxidation. The copper (I) 7-N-Etppz complex and H_2_O_2_ are required to produce hydrolysis products of acetonitrile (Supplementary Table 4, entries 1–5). With the copper salt and addition of hydrogen peroxide without the ligand there is some acetonitrile hydrolysis and minimal acetic acid production. Water is incapable of carrying out the hydrolysis. Among copper precursors, BF_4_ salt produces the most active complex in hydrolyzing MeCN (Supplementary Table 4, entries 5–7). The limiting reagent is H_2_O_2_ and the highly concentrated complex solution results in a lower TON (Supplementary Table 4, entries 8–11). The rate of addition (Table 2) and the amount of H_2_O_2_ impact the reaction exemplified by two different MeCN:H_2_O_2_ ratios (10:25 vs 57.5:1 Supplementary Table 4, entries 5,10). The cumulative turnover for MeCN hydrolysis of 1.6 was achieved with the MeCN:H_2_O_2_ molar ratio of 2 and reaction time of 24 h (Supplementary Table 4, entry 11). To examine the extent of complex activity towards hydrolyzing acetonitrile, we performed reactions with high H_2_O_2_ to acetonitrile ratio which led to significantly higher amounts of acetamide and acetic acid products (Supplementary Table 4, entry 10–11).

**Figure 2:**
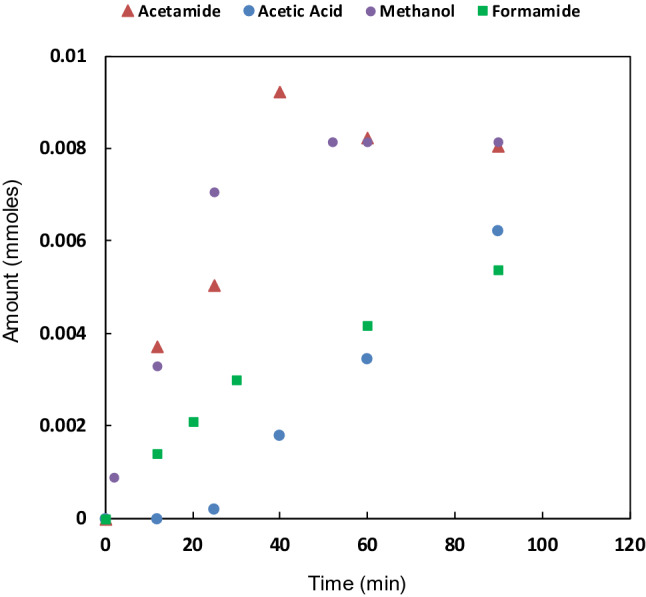
Time dependent acetamide and acetic acid production (Fig. 1) by complex 7-N-Etppz using BF_4_ based salt^a^. Due to low concentration, there is moderate uncertainty in acetic acid quantification. ^a^Reaction Conditions: The complex (40 mM), H_2_O_2_ (5 mmol), acetonitrile (1.0 mL) room temperature.

Figure 2 shows that initially, the rate of acetonitrile to acetamide oxidation is faster than the subsequent amide to acid hydrolysis. At a certain point the acetamide concentration decreases due to hydrolysis to acetic acid. The nitrile decomposition reaction ceases after about two hours due to complex deactivation and hydrogen peroxide degradation (Supplementary Fig. 10). Nitrile decomposition for a variety of nitrile compounds is illustrated by Table 1.

**Table 1:** Nitrile hydrolysis to amide by copper complex.

Entry	Substrate concentration (M)	Substrate	Products	H_2_O_2_ conversion %^a^	TON
1	10			90	25
2^b^	18			> 99	25
3	9.7			0	0
4	15			0	0
5^c^	18			25	7
6^d^	9			1	0.3
7	19			2	0.5

Reaction conditions: Each entry includes 57.5 eq (57.5 mmoles) of nitriles (except 2 and 6), H_2_O_2_ (1 mmol), Complex (BF_4_) (0.04 mmol, 4% of H_2_O_2_), time (2 h), room temperature.

^a^Conversion calculated based on oxidant (H_2_O_2_) as the limiting reagent.

^b^FCH_2_CN (30 mmol).

^c^Malononitrile melts at 32 °C, the reaction was carried out at 35 °C.

^d^Adiponitrile (15 mmol).

Substrate scope analysis was performed with high nitrile concentration and limited amounts of hydrogen peroxide. The trichloroacetonitrile and fluoroacetonitrile led to highest turnovers of nitrile to amide (Table 1, entries 1–2). Due to the presence of electron withdrawing groups, the nitrile group is more vulnerable to nucleophilic approach. The conversions were calculated based on hydrogen peroxide transformation to hydrolysis products since that was the limiting reagent. Electron donating groups such as aryl and vinyl prevent the hydrolysis (Table 1, entry 3–4). Complex is more active towards malononitrile compared to acetonitrile, due to the presence of more electron withdrawing effect of CH_2_CN compared to H (Table 1, entry 5, 7). Greater H_2_O_2_ conversion and TON are indicative of higher catalytic activity of malononitrile substrate compared to acetonitrile (Table 1, entries 5, 7). The further the nitrile groups are apart, the less they affect one another in the case of adiponitrile (Table 1, entry 6). Methanol production is summarized in Table 2. Hydrogen peroxide is better utilized when added slowly due to controlled degradation rate. When urea adduct of hydrogen peroxide was used, higher activity was achieved due to slow dissolution process of H_2_O_2_ into the medium (Table 2, entry 1–2). When added continuously with a rate close to acetonitrile hydrolysis, the complex activity is increased towards methanol production and acetonitrile hydrolysis (Table 2, entries 3–4). Bringing down the addition rate does not improve the activity except slightly (Table 2, entry 5).

**Table 2:** Complex activity towards methanol production regenerated by hydrogen peroxide addition rate.

Entry	Catalyst	H_2_O_2_ addition rate (mmol min^−1^)	Methanol (TON)	Amide (TON)	Carboxylic acid (TON)
1	Cu_3_L (BF_4_ source)^a^	NA^a^	0.2	0.4	0.4
2	Cu_3_L (BF_4_ source)^a,b^	NA^a,b^	0.35	NA	NA
3	Cu_3_L (BF_4_ source)	1	1.3	0.7	0.3
4	Cu_3_L (Acetate source)	1	1.3	2.2	NA^c^
5	Cu_3_L (ClO_4_ source)	0.1	1.4	0.9	0.7

Reaction conditions: Copper complex 0.04 mmol(1.3% of total H_2_O_2_), acetonitrile 18 eq (57.5 mmol), methane 14 eq (42 mmol), pressure 1 atm, H_2_O_2_ total 1 eq (3 mmol).

^a^Addition of H_2_O_2_ (all at once) instead of continuous.

^b^Urea adduct of hydrogen peroxide used instead of liquid solution, copper complex 0.04 mmol (5% of total H_2_O_2_), 0.8 mmol of hydrogen peroxide added.

^c^Accurate measurement not applicable due to overlap with acetate anion.

## Discussion

ESI–MS results imply the existence of a variety of complex species. A more prevalent presence of di and tricopper species is observed with perchlorate and acetate precursor compared to the tetrafluoroborate salt. This observation implies an interaction between the complex and counter anions^43^. Electronic absorbance spectra exhibits a 265–300 nm peak which is assigned to “oxide”-to-Cu^II^ LMCT^41^. The absorbance is more prominent for ClO_4_ and acetate based species where the tricopper species are more abundant. The considerably larger rate of H_2_O_2_ decomposition compared to the rate of acetamide production suggests that only about 10% of H_2_O_2_ is being utilized in the reaction. We have directionally showed by doing slow addition one can improve the overall TON for methanol. Isotope labeled experiment in presence of ^18^O_2_ showed some ^18^O incorporation in the acetamide compared to when complex is activated by natural isotope O_2_. Experiment with ^18^O labeled H_2_O_2_ also showed incorporation of ^18^O in the acetamide (Supplementary Fig. 11). This could be due to the stoichiometric reaction of Cu_3_LO_2_ making methanol. Addition of unlabelled H_2_O_2_ takes over in the subsequent steps of catalytic turn overs. We determined that produced methanol is at least partly supplied by solvent when acetonitrile was used. Reaction in absence of methane led to some methanol production and ^13^C labeled acetonitrile used as the solvent led to ^13^C methanol (Supplementary Fig. 12). We have observed exclusive formation of Cl_3_CONH_2_ and FCH_2_CONH_2_ instead of any chlorinated or fluorinated alcohol when trichloroacetonitrile or fluoroacetonitrile were used as solvent.

As a part of interrogating the reaction mechanism, we have studied the catalyst decomposition pathway. This aspect of the chemistry was not probed previously, and we recognize this issue as a key problem for this catalytic system. The active life-time of the complex is affected by a Cu leaching mechanism that eventually makes Cu(NH_3_)_x_ species. We believe ammonia evolution occurs through amide hydrolysis. Evidence of ammonia, water, and acetonitrile coordinated copper is present in the DART-TOF spectra in the range of 80–98 *m/z* (Supplementary Fig. 13) and ESI–MS (Supplementary Fig. 14). In the example of trichloroacetonitrile where the TON is high, there was no carboxylic acid detected in the product. This implies that subsequent hydrolysis of amide to acid is correlated with deactivation of the complex. However, due to poor solubility of complex (and possibly methane) into the solvent the TON for methane to methanol conversion is only modestly improved. Overall, we find that acetonitrile is not an innocent solvent in the peroxide mediated catalytic partial oxidation of methane to methanol using the tricopper complex. For the 7-N-Etppz copper complex to form tricopper species, the role of the counter anion needs to be considered since by changing this species, different spectroscopic and activity results are obtained. The extent of nitrile decomposition is controlled by the peroxide amount and the rate of addition as well as electron withdrawing groups attached to the nitrile. At least 90% conversion of peroxide (TON of 25) to amide is achieved in the case of trichloroacetonitrile and a TON of 1.6 was observed for the case of acetonitrile conversion to acetamide and acetic acid. When used as a medium for methane to methanol conversion, chlorinated acetonitrile led to a slightly higher TON of complex activity partly due to faster reactive oxygen species generation but was hindered by the lack of solubility of the complex in the medium.

## Methods

### Materials

All chemicals were purchased from Sigma-Aldrich unless mentioned otherwise. Solvents (Methanol and Acetonitrile) were first subject to continuous bed drying by solvent dispenser system under argon. They were subject to a freeze pump thaw procedure to remove residual oxygen content and finally were subject to 48 h of static drying by 3 A molecular sieves under argon and subsequent filtration prior to use. The precursors and solvents were stored under argon atmosphere.

### 7-N-Etppz ligand synthesis

The ligand was synthesized according to procedures introduced by Chan et al^3^. 15 mL of a 1.3 M solution of epichlorohydrin in methanol was added slowly to a 0.33 M solution of homopiperazine while stirred at − 5 °C. After 72 h the solution was briefly subject to rotary evaporation at room temperature to remove most of the solvent. 4.3 g of the resulting compound was added into a solution with 15 mL of acetonitrile, 4.15 g of K_2_CO_3_ and 3.5 g of 1-ethylpiperazine and heated to 80 °C for 48 h under argon atmosphere. The solution was then filtered and dried via a rotary evaporator at room temperature and stored under argon atmosphere. The H-NMR, and C-NMR characterization of the resulting ligand is provided in the SI. The NMR spectra (Supplementary Fig. 1) is consistent with previously reported NMR of the ligand. The ESI–MS characterization of ligand showed the major molecular ion peak at *m/z* 441.

#### Copper complex preparations

In a typical run for a 13 mM solution of complex in acetonitrile, 18 mg of 7-N-Etppz ligand and 36 mg of tetrakis(acetonitrile)copper(I) tetrafluoroborate were added to 3 mL of acetonitrile and stirred for desired amount of time prior to use.

#### Acetonitrile to acetic acid and acetamide transformation

In a typical run after the complex preparation within glovebox in a biotage sealed cap vial, up to 0.1 mL of a 35% (w/w) H_2_O_2_ solution was added to the solution and left stirring while taking samples at desired time intervals. The reaction is complete within the first 2 h.

#### Continuous hydrogen peroxide flow for methane to methanol conversion

In a typical run 0.01 mol per min of hydrogen peroxide was added to a 35 mL glass bottle with 3 mL of acetonitrile, catalyst concentration of 13 mM, and methane pressure of slightly above 1 atm.

#### Characterizations

^1^H and ^13^C NMR were performed with CDCl_3_ with a Bruker AVANCE 300 MHz. Electrospray Ionization Mass Spectrometry (ESI–MS) characterization of the complex was done by diluting complex solutions 1000 times from the original 13 mM to reach acceptable limits for use of a triple quadropole Quattro-II (Waters) instrument for low resolution analysis. For high resolution analysis a QStar Elite (AB sciex) instrument was used. Electron Paramagnetic Resonance (EPR) analysis was carried out on a Bruker instrument at 110 K, with a microwave frequency of 9.39 GHz and a modulation amplitude of 10 G. Ultraviolet–visible spectra of samples were obtained with a Shimadzu UV-2450 ultraviolet–visible spectrophotometer. Samples were exposed to air before UV–Vis spectrometer measurements.

## Supplementary Information

Supplementary Information.
