# Supplementary material for: Role of catalytic nitrile decomposition in tricopper complex mediated direct partial oxidation of methane to methanol
Source: Sci Rep. 2021 Sep 28;11:19175. doi: 10.1038/s41598-021-98721-2 (PMC8478979; doi:10.1038/s41598-021-98721-2)
Supplement: Supplementary file 1 — Supplementary Information. [file 41598_2021_98721_MOESM1_ESM.docx]

**Role of catalytic nitrile decomposition in tricopper complex mediated direct partial oxidation of methane to methanol**

Ehsan Moharreri,^1^ Tahereh Jafari,^1^ Dinithi Rathnayake, ^1^ Harshul Khanna, ^1^ Chung-Hao Kuo, ^1^ Steven L. Suib, ^1^ Partha Nandi^2*^

^1^Department of Chemistry and Institute of Materials Science, University of Connecticut, Storrs, CT 06269

^2^Corporate Strategic Research, ExxonMobil Research and Engineering, 1545 US 22 East, Annandale, NJ 08801

**Supporting Information**

**Content**

Synthesized 7-N-Etppz ligand (H-NMR and C-NMR) ………………………………………………………….2

Copper incorporation into complex (ESI-MS)…………………………………………………………………….3

Spectroscopy (UV-Vis, EPR, and Cu-NMR)………………………………………………………………………….6

Hydrogen peroxide decomposition (time dependent study)……………………………………………..12

18O labeling experiment (MS)…………………………………………………………………………………………..13

Cu leaching (DART-MS and ESI-MS)…………………………………………………………………………………..14

ESI summary tables…………………………………………………………………………………………………………..15

Spectroscopy summary tables…………………………………………………………………………………………..15

TON table for amide production……………………………………………………………………………………….16


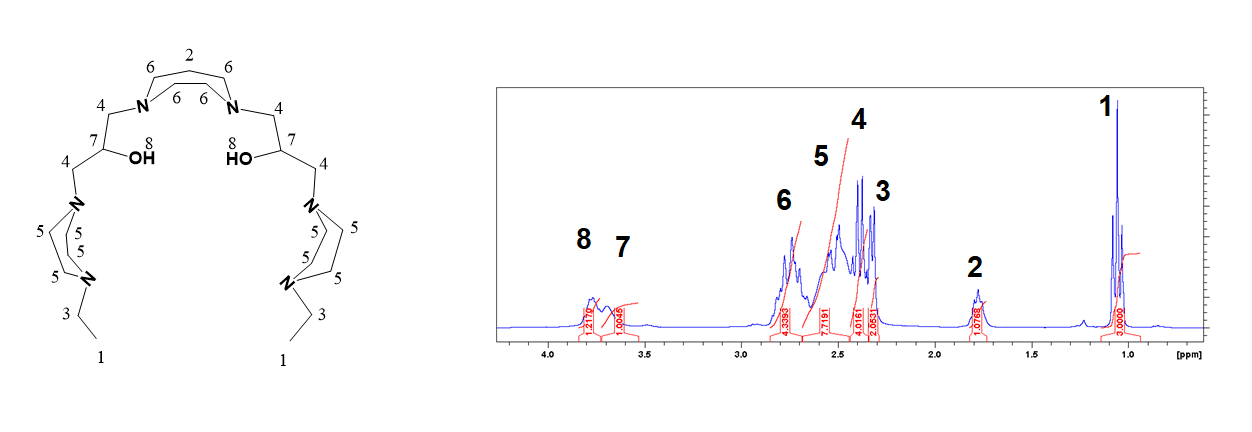


1H-NMR


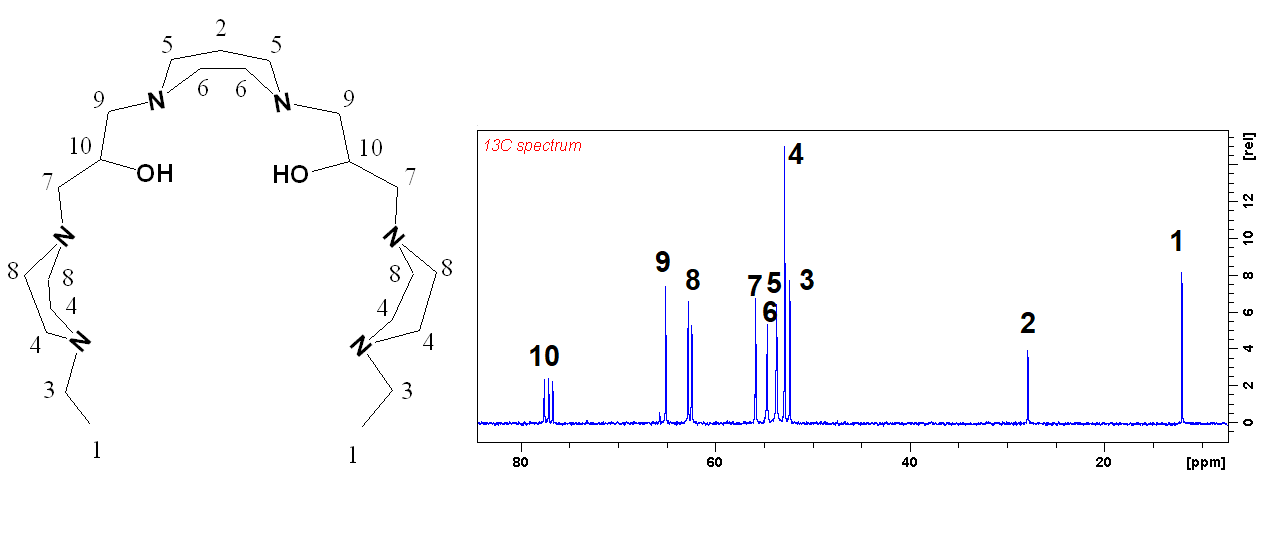


13C-NMR

**Supplementary Fig. 1**- H-NMR and C-NMR spectra of ligand in CDCl_3_.

ESI-MS

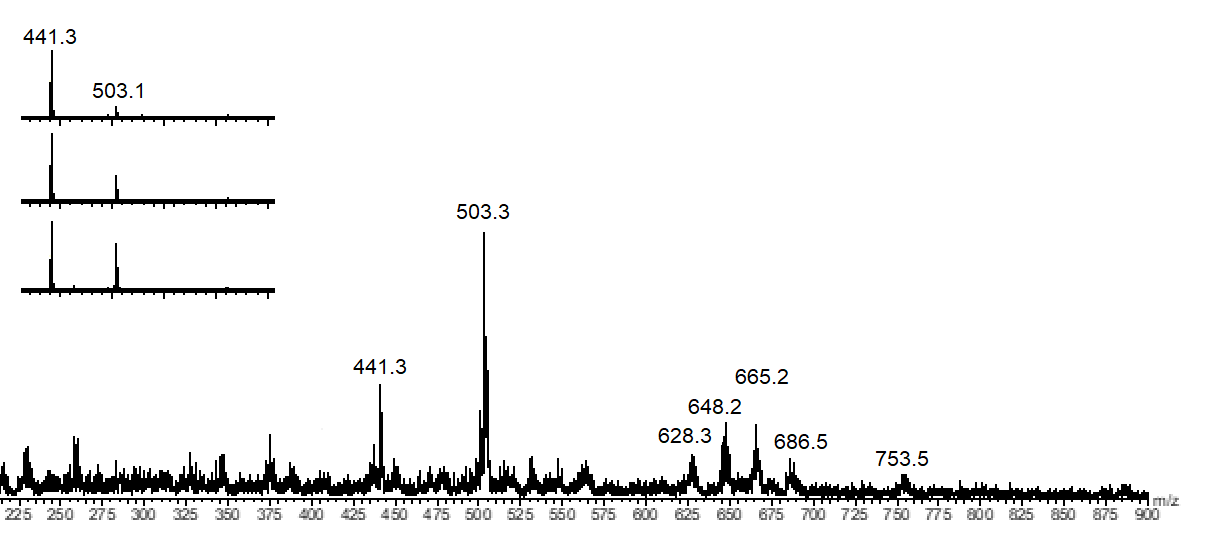


(a) 5 min

(b) 45 min

(c) 75 min

(d) 90 min

**Supplementary Fig. 2** ESI-MS stack plot showing complex formation by time with Cu(MeCN)_4_BF_4_ + 7-N-Etppz Ligand in a 13mM concentration. Supplementary Fig. 2 Increase of mono copper specie with time with BF4 precursor


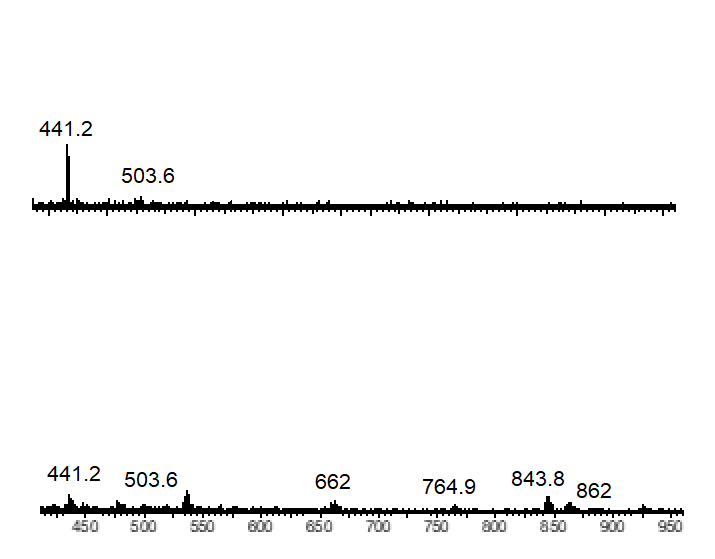


**(b) Cu: Ligand = 3**

**(a) Cu: Ligand = 2**

**Supplementary Fig. 3** ESI-MS stack plot showing complex formation by copper to ligand ratio with Cu(ClO_4_)_2_ precursor and 7-N-Etppz Ligand after 75 minutes

.

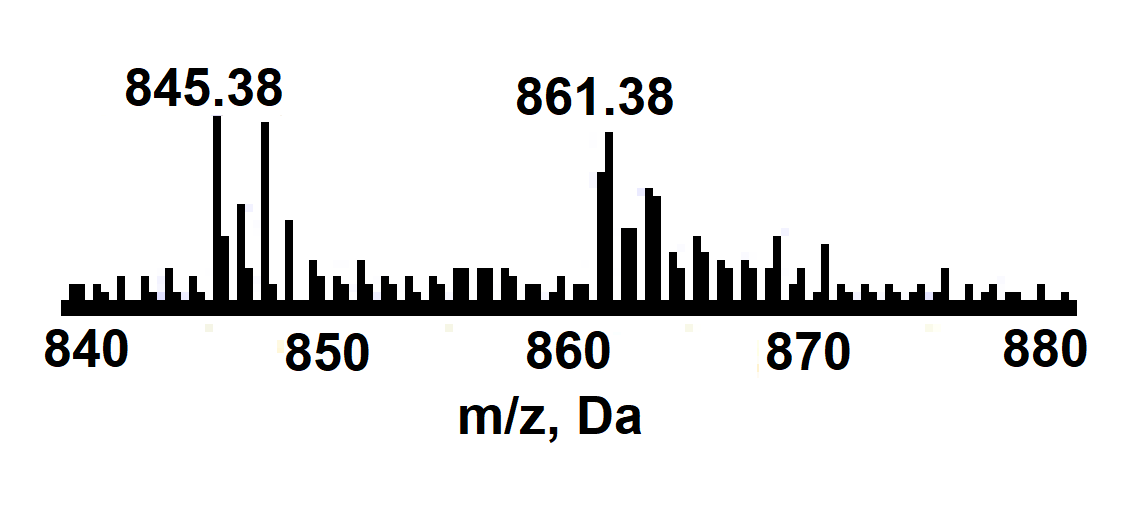


**Supplementary Fig. 4** High Resolution ESI-MS of the complex showing the presence of attached acetonitrile and water molecules. Acetonitrile and acetamide molecules are coordinating with the coppers.

UV-VIS


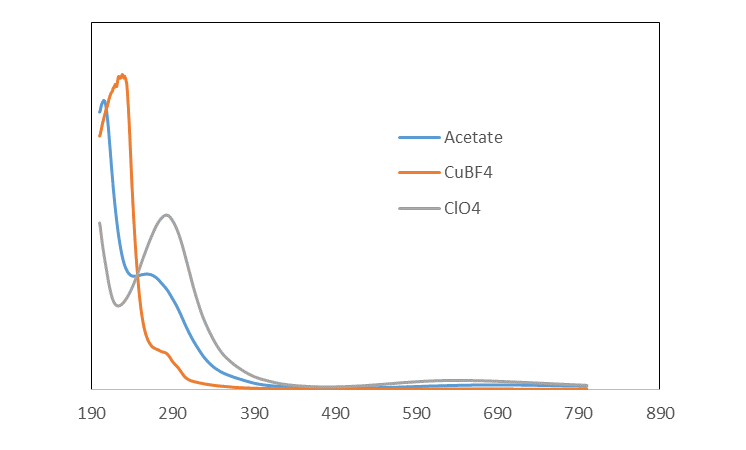


**“oxide”-to-Cu^II^ LMCT**


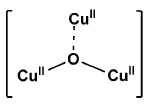

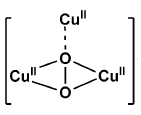


**Ligand field**

**transitions of Cu^II^**

**265 nm**

**380 nm**

660 nm


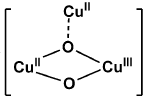


**300 nm**

Acetate

BF_4_

ClO_4_

**peroxo-to-Cu^II^**

**Supplementary Fig. 5** Electronic absorption spectra with assigned tricopper species for various counteranion salts.

**Cu_3_LO_2_**

**Cu_3_LO_2_ + H_2_O_2_**

**Supplementary Fig. 6** Electronic absorption spectra with assigned tricopper for ClO_4_ based species before and after addition of H_2_O_2_ highlighting the decrease of the oxide-to-Cu^II^ LMCT.

**Supplementary Fig. 7** EPR spectra of BF_4_ salt based complex in acetonitrile at 110 K

**Supplementary Fig. 8** EPR spectra of Acetate salt based complex in acetonitrile at 110 K

**Supplementary Fig. 9** EPR spectra of ClO_4_ salt based complex in acetonitrile at 110 K

63 CuNMR


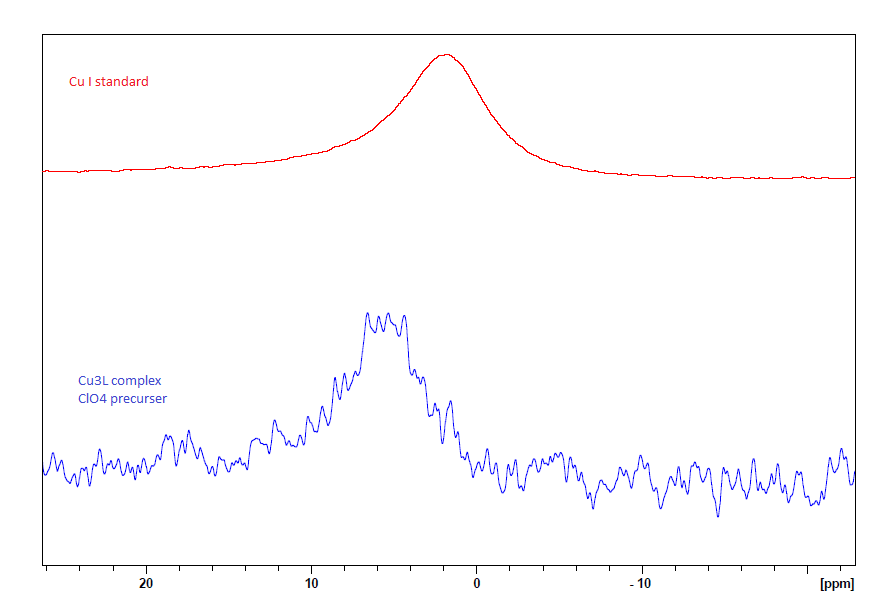


**Supplementary Fig. 10** 63 Cu NMR spectra of 7-N-Etppz Complex by ClO_4_ based salt in deuterated acetonitrile (blue), Copper (I) standard solution (red).

**Supplementary Fig. 11** H_2_O_2_ decomposition with 7-N-Etppz Complex by BF_4_ based salt in acetonitrile. This corresponds to reaction conditions of entry 5 of **Supplementary Table 4**.


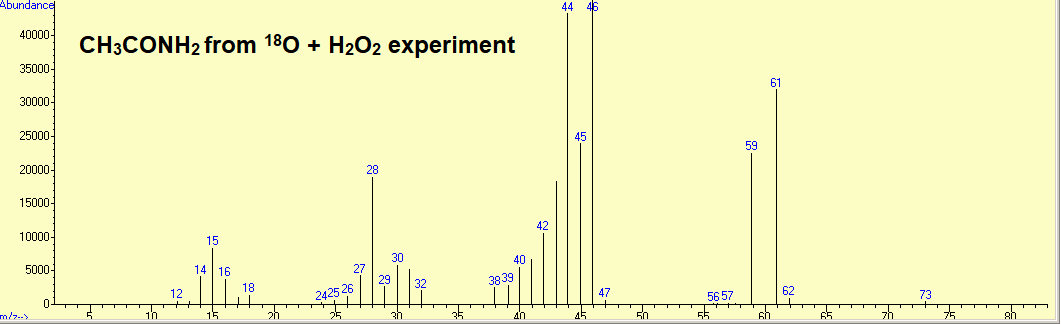


2

**(a)**


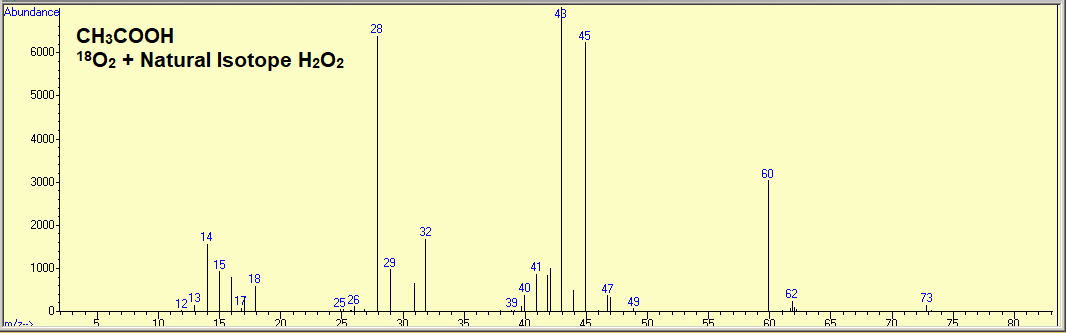


**(b)**

**(c)**

**
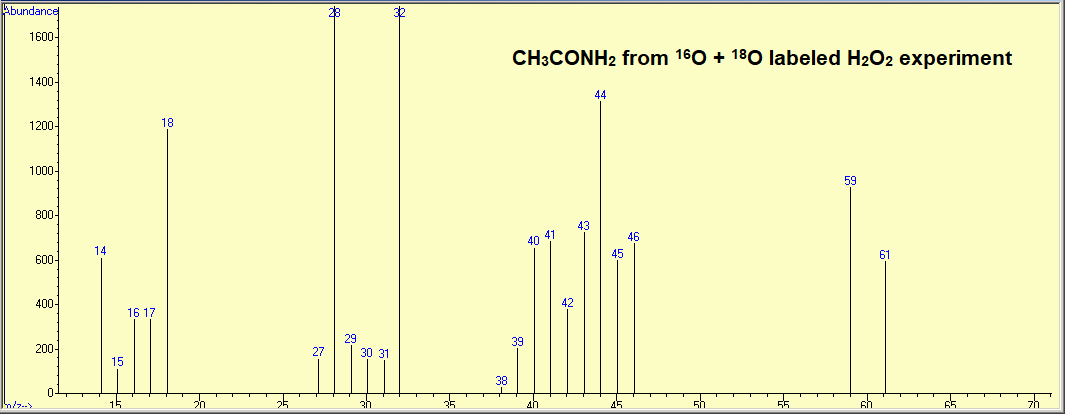
Supplementary Fig. 12** (a) Mass spectrum of acetamide when reaction occurrs in ^18^O presence. (b) Mass spectrum of acetic acid when reaction occurs in ^18^O presence (c) Mass spectrum of acetamide when reaction occurs in ^16^O presence with ^18^O labeled H_2_O_2_.

2

 **Supplementary Fig. 13** DART-TOF of copper complex based on BF_4_ salt after addition of H_2_O_2_ and 2 h of stirring.


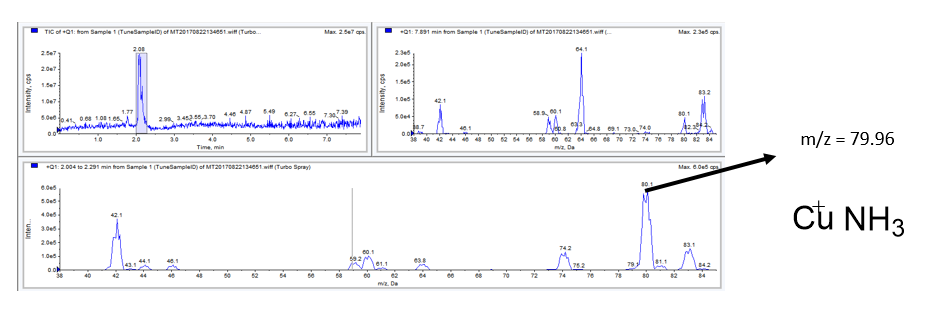


**Supplementary Fig. 14** ESI-MS of copper complex based on BF_4_ salt after addition of H_2_O_2_ and 2 h of stirring

| **Complex** | **MonoCopper Species (m/z)** | **Dicopper Species (m/z)** | **Tricopper Species (m/z)** | **Other peaks (m/z)** |
| --- | --- | --- | --- | --- |
| Cu:L (BF_4_) | 503 | ----- | ---- | 441 |
| Cu:L (ClO_4_) | 503 | 662 | 764,843 | 375 |
| Cu:L (Acetate) | 562 | 564, 806 | 867, 686, 882 | 479,622 |

**Supplementary Table 1** Copper species identified by ESI-MS varied by precursor type.

| **Complex** | **(^63^Cu NMR) (ppm)** | **g factor (EPR)** | **UV-Vis λ nm, (ε M^-1^cm^-1^)** |
| --- | --- | --- | --- |
| Cu:L (BF_4_) | ----- | ----- | λ_max_=230(10000), 290^s^ |
| Cu:L (ClO_4_) | 5 | 2.12 | λ_max_=290(9000), 650^L^ |
| Cu:L (Acetate) | --- | 2.13 | λ_max_=210(5000), 280^s^, 690^L^ |

**Supplementary Table 2** Copper species identified by ^63^Cu-NMR, EPR (as prepared) and UV-VIS (oxidized) adsorption varied by precursor type. NMR and EPR values presented here correspond to as prepared complex meaning they were not exposed to air. The UV-Vis peaks in the table were measured on the air exposed (oxidized) complex. ^s^Shoulder UV-vis peak. ^L^Ligand field interaction peak.

| **Entry** | **As prepared** | **Oxidized** | **With H_2_O_2_** |
| --- | --- | --- | --- |
| Cu:L (BF_4_) | -----^a^ | 2.07, 2.10 | 2.07, 2.11 |
| Cu:L (ClO_4_) | 2.12 | 2.12 | 2.11 |
| Cu:L (Acetate) | 2.13 | 2.13 | 2.08, 2.10 |

**Supplementary Table 3** Copper species g factor (EPR) varied by precursor type, presence of oxygen and hydrogen peroxide.^a^ No clear signal was detected for as prepared complex with BF_4_ based precursor.^b^ Some weak hyperfine coupling features were detected

| **Entry** | **Catalyst** | **C_catalyst_ (mM)** | **Products** | | | |
| --- | --- | --- | --- | --- | --- | --- |
|  |  |  | **CH_3_COOH**  **Acetic Acid (TON)** | **CH_3_CONH_2_**  **Acetamide (TON)** | **CH_3_COOH**  **Acetic Acid (mM)** | **CH_3_CONH_2_**  **Acetamide (mM)** |
| 1 | None | 0 | 0 | 0 | 0 | 0 |
| 2 | BF_4_ copper salt | 13 | 0.05 | 0.1 | 0 | 1 |
| 3 | Complex (BF_4_)^b^ | 13 | 0 | 0 | 0 | 0 |
| 4 | EP-Copper Complex (BF_4_)^c^ | 13 | 0 | 0.1 | 0 | 1 |
| 5 | Complex (BF_4_) | 13 | 0.4 | 0.4 | 5 | 5 |
| 6 | Complex(ClO_4_) | 13 | 0.1 | 0.1 | 1.7 | 1.5 |
| 7 | Complex(Acetate) | 13 | - | 0.1 | - | 1 |
| 8 | Complex (BF_4_) | 40 | 0.1 | 0.1 | 3 | 1 |
| 9 | Complex (BF_4_) | 80 | 0.03 | 0.1 | 3 | 1 |
| 10 | Complex (BF_4_)^d^ | 13 | 0.6 | 0.6 | 8 | 8 |
| 11 | Complex (BF_4_)^e^ | 36 | 0.5 | 1.1 | 17 | 39 |

**Supplementary Table 4** Transformation of acetonitrile to acetic acid and acetamide by copper complex with various copper salts and 7-N-Etppz Ligand^a^. ^a^**Reaction Conditions:** Acetonitrile (3 mL, 57.5 mmol, 19M), H_2_O_2_ (1 mmol), time (2 h), room temperature. ^b^ Water (1mmol) instead of H_2_O_2_. ^c^Copper and 1-Etppz instead of the ligand with a 1:1 molar ratio. ^d^ H_2_O_2_ (25 mmol), acetonitrile (10 mmol). ^e^ H_2_O_2_ (5 mmol), acetonitrile (10 mmol), analyzed after 24 hours. Limited amounts of formamide and N-ethylformamide byproducts are formed in a typical reaction involving these systems.
